# A Numerical and Experimental Study on a Pre-Twisted Ring Spinning System

**DOI:** 10.3390/polym10060671

**Published:** 2018-06-16

**Authors:** Keyi Wang, Wenliang Xue, Longdi Cheng

**Affiliations:** 1 Key Laboratory of Textile Science & Technology, Ministry of Education, Donghua University, Shanghai 201620, China; kywang@dhu.edu.cn (K.W.); xwl@dhu.edu.cn (W.X.); 2 College of Textiles, Donghua University, Shanghai 201620, China

**Keywords:** ring spinning, pre-twister, CFD, nozzle, productivity, yarn properties

## Abstract

The ring spinning process is the most widely used method in the spinning industry. Nowadays, the labor cost become more and more expensive, and it is essential to improve productivity. For increasing the productivity, a modification of adding a pre-twister and holding roller on the traditional ring spinning system have been discussed in this paper. The computational fluid dynamics (CFD) are introduced to study the effects of pre-twister and spinning tests are implemented for verification. The numerical simulations show that the cavity conical degree and nozzle numbers of the pre-twister are the key parameters which affect the airflow fluctuation in the cavity, and have obvious effects on the resultant yarn twist. By contrast, the axial angle and tangential angle of the nozzle have less effect on the resultant yarn twist. When the fiber bundles pass by the front nip, they are affected by the vortex and result in a partially strengthened and wrapped structure which could be subsequently twisted less by the traveler and ring, so the productivity could be potentially increased. According to the spinning tests, an evident productivity increase by nearly 30% for medium cotton yarns can be achieved, and the yarns have an acceptable reduction in nearly all properties.

## 1. Introduction

Staple fiber spinning has a long history of more than 20,000 years [1]. Keeping in pace with scientific technologies, many new open-end and non-open-end spinning systems such as rotor spinning, air-jet spinning, vortex spinning, and self-twist spinning have appeared in succession. These new technologies have led to increases in spinning speed and, subsequently, production [2]. However, the ring spinning method is still the most widely used in today’s textiles mills due to the high quality of the yarn, being both finer and stronger than the yarn produced by open-end and non-open-end spinning systems [3]. With the increasing cost of labor in recent years, the importance of increasing spinning productivity through technological improvements is evident.

In order to achieve higher productivity, many developments have occurred that are intended to increase the spindle speed, or to reduce or eliminate the friction between ring and traveler [4,5,6,7,8,9,10,11]. However, these developments have improved the ring spinning productivity in a very limited range or have not yet been implemented in industry due to their complexity.

For the purpose of improving ring spinning productivity, Sawhney and Kimmel [12] worked on a “air-plus-ring” tandem spinning system, which could produce a low-twist, novel ring yarn at a relatively high speed. Their preliminary investigations showed that the yarn was weaker than a comparable ring-spun yarn although spinning speeds for certain yarns may be up to 50% higher than those prevalent in conventional ring spinning. No further research has been reported since then. 

Many researchers have worked on yarn formation theory focusing on the airflow characteristics. In the earlier reports, Kong and Platfoot [13] studied the two-dimensional simulation of airflow in the transfer channel of rotor spinning. Zeng and Yu [14,15] studied the airflow in the first and second nozzle of air-jet spinning from two-dimensional to three-dimensional simulation. Some of the researchers [16,17,18] studied the principle of yarn formation of Murata vortex spinning, also by airflow simulation. Bergada et al. [19] studied the flow characterization in cylinders for pneumatic spinning and got a clear understanding of the vortex with extensive CFD (computational fluid dynamics) simulation of the airflow. Zou et al. [20] studied the twisted strength of the whirled airflow in Murata vortex spinning by an analytical model based on simulating the flow field inside the nozzle block. Rengasamy et al. [21,22] studied the airflow in nozzle-ring spinning by CFD to explain the principle of reduction in yarn hairiness.

In this paper, we introduce the model of a pre-twister spinning system, which could produce pneumatically entangled real-twists on the fine strand prior to the twisting by the ring/traveler system. Less twist will be added to the yarn in the subsequent process, and the productivity can be increased. The design of the pre-twister is investigated through the airflow characteristics with CFD. The spinning tests are carried out for verification and the yarn properties are compared with those of classic ring-spun yarn.

## 2. Model of the Spinning System and the Pre-Twister 

**Figure 1:**
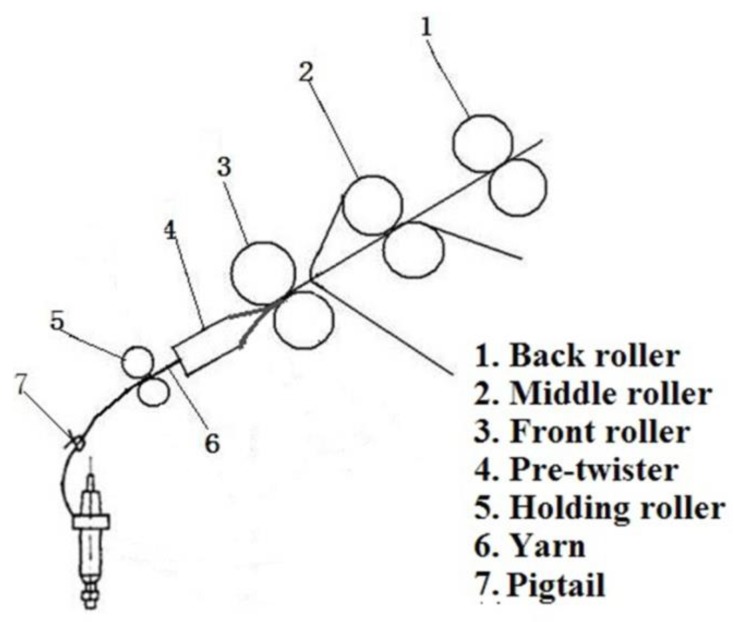
Schematic diagram of the spinning system.

The model of the modified ring spinning system is shown in Figure 1. Compared with the conventional ring spinning system, a pre-twister and a holding roller were added to the system, which is also different from the former researchers [21,22,23,24,25] whose purposes were reducing yarn hairiness with an air-jet and without a holding roller.

**Figure 2:**
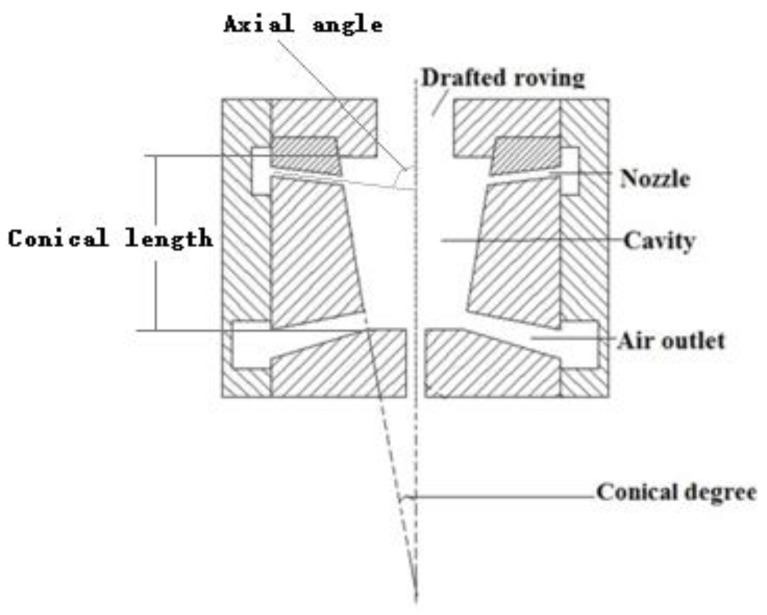
Cross-sectional view of the pre-twister.

In this model, the pre-twister is located very close to the front roller nip. After draft of the roving, the strand of fibers from the nip of the front roller is fed into the pre-twister where the air is compressed from the 4 nozzles, as shown in Figure 2.

## 3. Numerical Simulation of Pre-Twister

The key to this supposed spinning system is the design of the pre-twister. The airflow pattern inside the pre-twister was analyzed and simulated using a fluid flow analysis package, ANSYS Fluent14.0.0 (ANSYS, Canonsburg, USA), so as to optimize the design of the pre-twister.

According to the calculation of fluid mechanics in the Reynolds number,

(1) $$R_{e}=\rho \mu D/\mu$$

where ρ is the air density (kg/m^3^), *u* is the air velocity (m/s), *D* is the inner diameter of the pre-twister, and μ is the viscosity of air (N·s/m^2^). The Reynolds number of the airflow in the pre-twister is in the order of 10^5^, and the airflow is classified into turbulence. In order to simplify the calculation, we considered that the airflow was not affected by the fiber and the air was considered as viscous and compressible. Regardless of heat exchange, the Realizable k–ε Model was used for constant enthalpy flow.

The basic geometry of the pre-twister is a cavity with 4 nozzles as shown in Figure 2. The cavity shape is conical with 6 mm length. The nozzles are conical in shape with 0.8 mm × 0.6 mm diameter and 2 mm length connected to the cavity wall. The axial angle α is the angle between the nozzle axis and the cavity axis and the tangential angle β is the angle between the nozzle axis and the cavity wall. The conical degree of the cavity and the axial angle, tangential angle, and number of nozzles were selected as geometry design parameters for computation. Nine geometries of the pre-twisters were chosen, as given in Table 1. 

For each case, the boundary conditions are set as follows: the inlet air pressure is 0.2 MPa (absolute) and the outlet air pressure equal to the external atmospheric pressure, the face space is 0.2 mm, and the wall is stationary. The computational grid is generated by a patch-independent tetrahedral grid and the computational domain contains about 200,000 cells for each case.

## 4. Spinning Experiment

**Figure 3:**
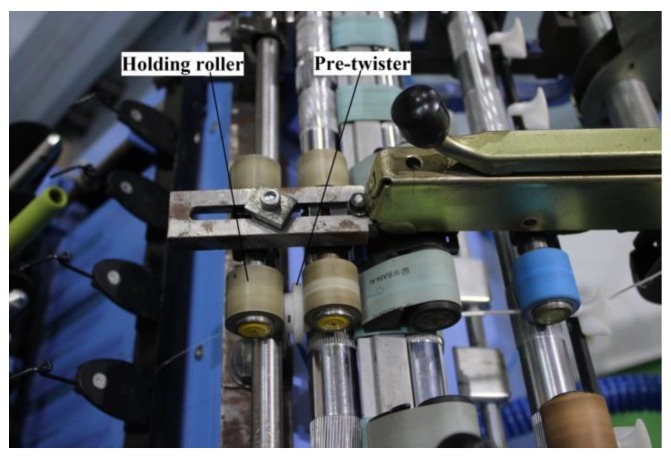
Modified Spintester.

In order to verify the model, we used a six-spindle lab Spintester operating in a standard textile lab to produce 15.3 tex spun yarns, employing (a) 370 tex carded cotton rovings, (b) the traditional traveler and optimum spindle speed of 10,000 rpm for the given 4.2 cm diameter ring, and (c) the standard yarn twist level (800 tpm). We then modified the Spintester by installing the pre-twister (Case1) located in the yarn path close to the front roller nip (Figure 3). We found that the nozzle air pressure from 0.2 to 0.3 MPa (absolute) was optimum for satisfactory spinning after multitests.

Except for a lower twist level (650 tpm), we employed the same rovings and other spinning conditions to produce 15.3 tex yarns on the modified Spintester under a reasonably acceptable level (less 40 ends down/1000 spindle hours).

The tensile properties of the yarns were measured on an USTER TENSORAPID (Uster, Uster, Switzerland) under a gauge length of 500 mm and a crosshead speed of 200 mm/min. The yarn evenness characteristics were tested on an USTER ME100 (Uster, Uster, Switzerland) with a 1000 m length of yarn and every test was performed at a speed of 200 m/min. The sensitivity settings used for counting thick, thin, and neps were +50%, −50%, and +200%, respectively.

Yarn samples were kept in the standard test environment (temperature: 20 ± 2 °C, humidity: 65 ± 4%) for 24 h prior to testing. Thirty readings were taken for each sample.

## 5. Results and Discussion

### 5.1. The Airflow Trace in the Pre-Twister

**Figure 4:**
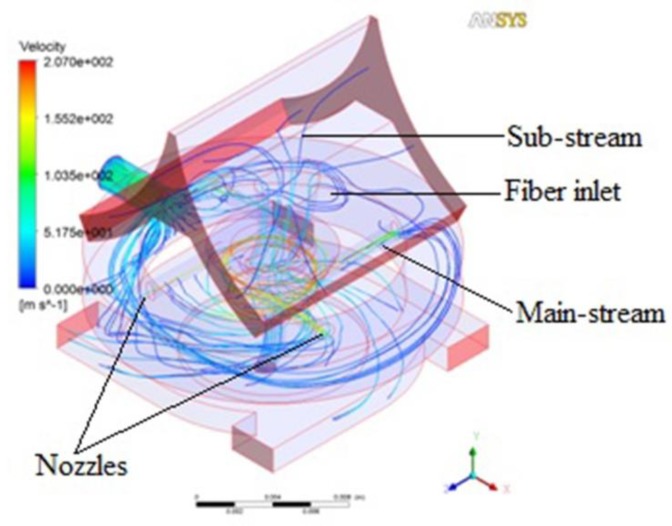
Airflow trace in the pre-twister.

The airflow in the pre-twister is mainly formed by two parts. The simulation of airflow trace is shown in Figure 4. One is the airflow compressed from the nozzles which moves forward spirally (main-stream). Another is the airflow that is sucked into the pre-twister from the fiber inlet (sub-stream), which moves downward axially. 

**Figure 5:**
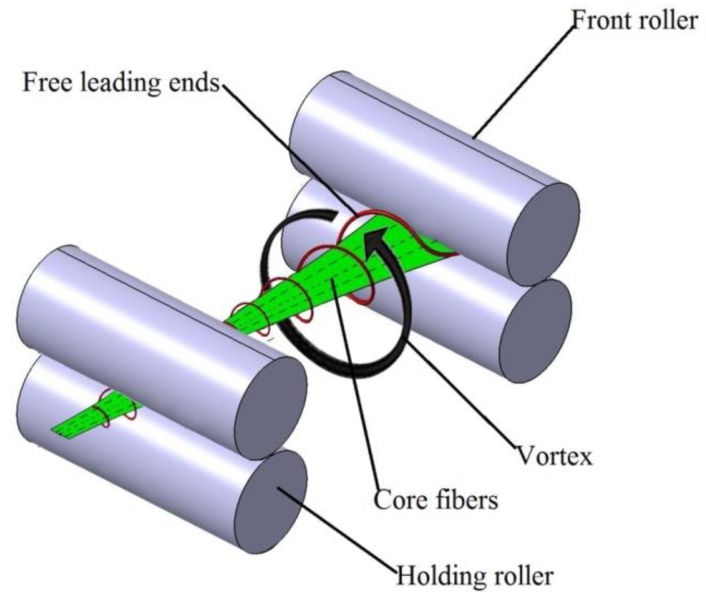
The pre-twisting process.

As the drafted fibers emerge from the front roller nip and are fed into the pre-twister, the leading ends of some marginal fibers are sucked into the pre-twister by the sub-stream while the other ends are held by the front nip; we call those leading ends the “free leading ends”. As shown in Figure 5, because the respective ends of the core fibers are firmly held by the front roller and holding roller, the free leading ends would be wrapped on core fibers with the help of the main-stream. With the entanglements by the free leading ends which are continuously controlled by the holding roller, the air vortex converts the drafted fibers into a partially strengthened and wrapped structure. This partially fabricated and strengthened yarn structure continues its uninterrupted journey to a conventional ring spinning zone, where it is finally and positively twisted (unidirectionally by the ring and traveler combination) into a novel yarn. This is a continuous process. Due to this reason, twists by the travelers would be decreased and productivity could potentially be increased.

**Figure 6:**
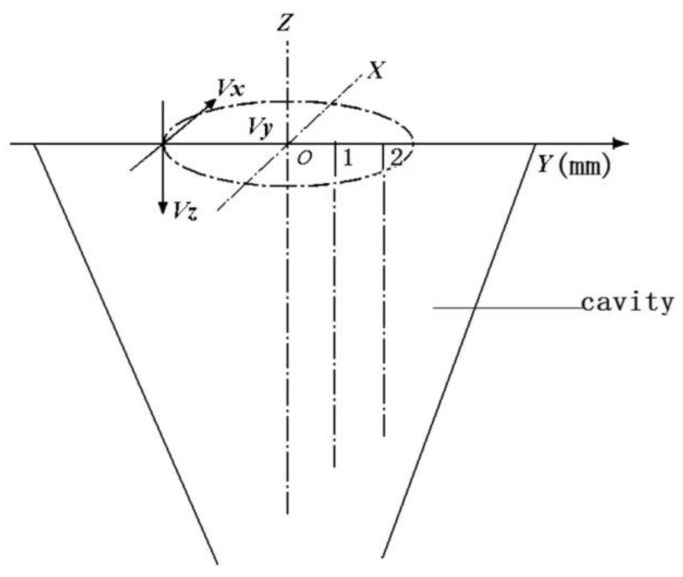
The definition of velocity in the pre-twister.

**Figure 7:**
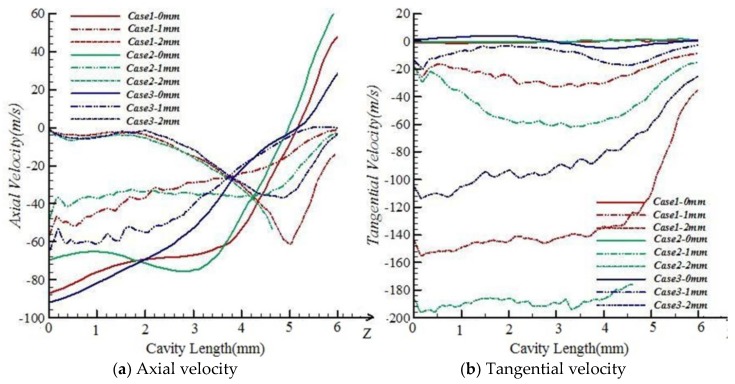
Velocity comparisons among Case1, Case2, and Case3.

**Figure 8:**
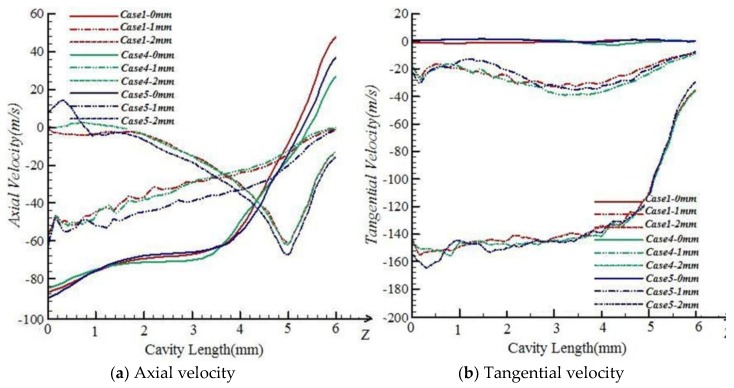
Velocity comparisons among Case1, Case4, and Case5.

**Figure 9:**
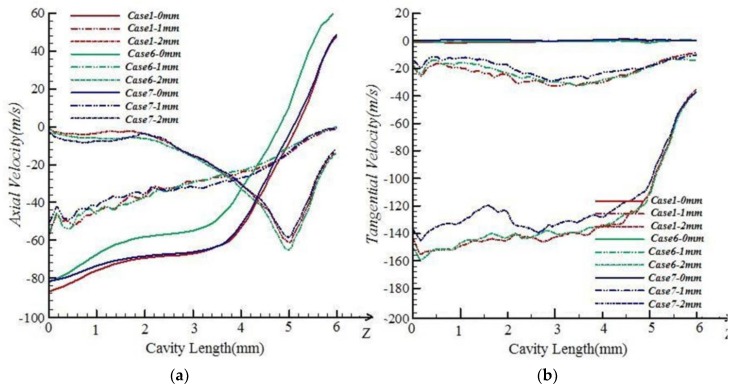
Velocity comparisons among Case1, Case 6, and Case 7. (**a**) Axial velocity; (**b**) tangential velocity.

**Figure 10:**
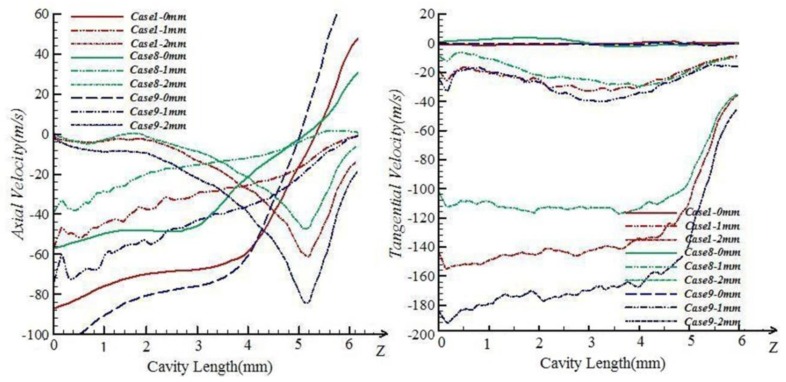
Velocity comparisons among Case1, Case8, and Case9.

The characteristics of the airflow in the pre-twister for each case in Table 1 were simulated, and are shown in Figures 7–10. The air field can be described with the airflow trace and its velocity which will directly influence the wrapping effect of the pre-twister. The air velocity is resolved into three components, viz., tangential velocity (V*_x_*), radial velocity (V*_y_*), and axial velocity (V*_z_*), as shown in Figure 6. Wrapping action is created by the tangential and axial velocity components of the air velocity. The total width of the drafted fibers emerging from the front roller nip is 2~4 mm (varied by the roving count), so the distance from core to marginal fiber is 1~2 mm. Thus, on the radial direction along the axis 1 and 2 mm away from it, the tangential and axial velocities from inlet to outlet are discussed.

#### 5.1.1. Effects of Conical Degree

Figure 7 shows that the tangential velocities in the axis along fiber inlet to outlet are not obviously affected by conical degree, but the tangential velocities in the positions of 1 and 2 mm away from the axis in Case2 are higher than those in Case1 and Case3. When the conical degree gets bigger, the tangential velocities in the positions away from the axis will be built up, which, in turn, allows the free leading ends to wrap on core fibers strongly. 

For axial velocities in the positions 1 and 2 mm away from the axis along fiber inlet to outlet, the variations caused by conical degree are not significant. When the conical degree gets bigger, the axial velocities in the axis will be smaller. The variations of axial velocities caused by conical degree have a slight effect on the entanglement of free leading ends.

#### 5.1.2. Effects of Nozzle Axial Angle α

As shown in Figure 8, the axial velocities and tangential velocities among Case1, Case4, and Case5 are nearly the same. Along the axis, the axial velocities close to the fiber inlet are all about 90 m/s. In the positions 1 and 2 mm away from the axis, tangential velocities along fiber inlet to outlet are also kept at the same level. It is noted that the nozzle axial angle α has a slight impact on the wrapping effect on the free leading ends.

#### 5.1.3. Effects of Nozzle Tangential Angle β

Figure 9 shows that among different cases, the variations in axial and tangential velocities in the cavity caused by the nozzle tangential angle β are similar to the conditions of the nozzle axial angle. When close to the fiber inlet, the axial velocities are all about 85 m/s. 

The tangential velocities in the positions 1 and 2 mm away from the axis along fiber inlet to outlet of Case7 are smaller than those of Case1 and Case6. In Case7, nozzle tangential angle β is set to 40°. Compared with β variation, the velocity’s variation is relatively small. The nozzle tangential angle also can be considered as a parameter which has a slight impact on the wrapping effect on the free leading ends.

#### 5.1.4. Effects of Nozzle Number

Figure 10 shows that the influence on airflow caused by number of nozzles is obvious. Tangential velocities in the positions 1 and 2 mm away from the axis along fiber inlet to outlet in the cavity of Case9 are much higher than those of Case1, and tangential velocities in the cavity of Case1 are much higher than those of Case8. The tangential velocities in the fiber inlet area are stronger with more nozzles. When close to the fiber inlet, tangential velocities and axial velocities are kept at the same level in all cases. When the nozzle number is increased, the wrapping effect on the free leading ends is stronger but may bring slight destruction to the parallel structure of core fibers. When energy saving is taken into consideration, the number of the nozzles must be controlled under 6 [26,27,28].

### 5.2. Yarn Testing Results

The yarn properties measured in terms of evenness, imperfections, and tensile properties are shown in Table 2. All properties of yarns produced on the modified Spintester are slightly worse than those of the classic ring-spun yarns. However, all typical properties of the modified spun yarn are generally satisfactory. Compared to the throughput spinning speeds of classic ring-spun yarns, the yarn productivity increase for the modified Spintester is 27.13%. Obviously, for the modified Spintester, the ability to produce a novel yarn with relatively low twist, which the traveler does, is the key to relatively higher yarn productivity. In this paper, we just conduct a preliminary investigation and this assumption may be a chance to improve the productivity of ring spinning.

## 6. Conclusions

According to the simulation and spinning tests mentioned above, a modified ring spinning system is introduced. For producing a low-twist and novel ring yarn at a relatively high speed, the drafted fibers from the front roller nip need to be wrapped and well controlled during pre-twisting. The wrapping degree of the free leading ends of fibers onto the core fibers will determine the result of the pre-twisting. Therefore, the airflow in the pre-twister is important. By numerical simulation, some parameters that are important to the airflow in the pre-twister are noted, such as the cavity conical degree and the nozzle number. When these parameters are changed, the airflow will be changed obviously. A stronger vortex can be reached with bigger conical degree, but the parallel structure of the core fibers will be destroyed if the conical degree is too big. A stronger vortex can also be reached with a higher number of nozzles. Increasing the number of nozzles also increases the consumption of energy. We suggest 4 to 6 nozzles on a pre-twister. Nine different cases of the pre-twister were investigated by computation, and Case1 was the optimized one. The spinning tests show that even though the yarn is weaker than classic ring-spun yarn, potentially higher yarn productivity is reached. This is expected to generate some commercial interest. Another advantage of this new spinning system is that it can be easily installed on existing ring spinning frames to maximize yarn productivity. When an optimized pre-twister is available, it will be a revolution in the spinning industry.

## Figures and Tables

**Table 1:** Characteristics of the investigated geometries.

		Case1	Case2	Case3	Case4	Case5	Case6	Case7	Case8	Case9
Cavity Conical Degree (°)		29.1	43.6	14.5	29.1	29.1	29.1	29.1	29.1	29.1
Nozzle	Axial Angle α (°)	10	10	10	30	50	10	10	10	10
	Tangential Angle β (°)	0	0	0	0	0	20	40	0	0
	Numbers	4	4	4	4	4	4	4	3	6

**Table 2:** Parameters and properties of yarns produced on the pure ring and modified Spintester.

Spinning Method	Ring Spun	Modified
Yarn size (tex)	15.3	15.3
Max yarn production rate Attained (m/min)	12.68	16.12
Increase in productivity over pure ring spinning (%)	N/A	27.13
Yarn twist level (tpm)	800	650
Tenacity (cN/tex)	20.66	18.02
C.V. of bkg. St.	9.6	10.5
Single-strand bkg. Elongation (%)	5.79	5.37
Unevenness, C.V. (%)	17.28	18.64
Thin places (−50%)	42.5	90
Thick places (+50%)	417.5	522.5
Neps (+200%)	565	655

## References

[1] Lawrence C.A. (2010). Advances in Yarn Spinning Technology.

[2] Wang S.Y., Yu X.Y. (2007). New Textile Yarns.

[3] Oxtoby E. (1987). Spun Yarn Technology.

[4] Hossain M., Abdkader A., Cherif C., Sparing M., Berger D., Fuchs G., Schutz L. (2014). Innovative Twisting Mechanism based on Superconducting Technology in a Ring-Spinning System. Text. Res. J..

[5] Ryohei K., Masamichi H., Naohumi K., Yoshihumi N. (2003). Spinning Ring for a Ring Spinning Machine and Method of Manufacturing Thereof. U.S. Patent.

[6] Faissal A., Yehia E. (2006). Ring Spinning System for Making Yarn Having a Magnetically Elevated Ring. U.S. Patent.

[7] Stalder H., Neff A., Oberholzer F. (1998). Ring Spinning Machine with Conical Rings. Germany Patent.

[8] Kubovy V., Blazek P., Didek S., Doležal J., Pavliček L., Plananský A., Šlingr J., Stejskal A. (2006). Machine for Loop-Spinning and Twisting. Germany Patent.

[9] Yashusi I. (2003). Spining Ring. Europe Patent.

[10] Helmut H. (2000). Ring-Traveller Combination of Ring Spinning and Twisting Machine. Germany Patent.

[11] Abdel-hady F., Mogahzy Y.E., AbuElenin S., Abdel-Kader R. (2006). Innovative approach to high-speed spinning using a magnetically-elevated spinning ring. AUTEX Res. J..

[12] Sawhney A.P.S., Kimmel L.B. (1997). Air and ring combination in tandem spinning. Text. Res. J..

[13] Kong L.X., Platfoot R.A. (1996). Two-dimensional simulation of air flow in the transfer channel of Open-End rotor spinning machines. Text. Res. J..

[14] Zeng Y.C., Yu C.W. (2003). Numerical simulation of air flow in the nozzle of an air-jet spinning machine. Text. Res. J..

[15] Zeng Y.C., Yu C.W. (2004). Numerical simulation of the fiber motion in the nozzle of an air-jet spinning machine. Text. Res. J..

[16] Guo H.F., Zeng Y.C., Yu C.W. (2010). Numerical study on the effect of geometric parameters of the second nozzle in air-jet spinning. J. Text. Inst..

[17] Pei Z., Yu C.W. (2009). Study on the principle of yarn formation of Murata vortex spinning using numerical simulation. Text. Res. J..

[18] Pei Z., Yu C.W. (2010). Numerical and experimental research on the influence of parameters on the tensile properties of Murata vortex yarn. J. Text. Inst..

[19] Bergada J.M., Valencial E., Coll L.L. (2007). Flow characterization in cylinders for pneumatic spinning. Text. Res. J..

[20] Zou Z., Cheng L., Xue W., Yu J. (2008). A Study of the twisted strength of the whirled airflow in Murata vortex spinning. Text. Res. J..

[21] Rengasamy R.S., Patnaik A., Anandjiwala R.D. (2008). Simulation of airflow in nozzle-ring spinning using computational fluid dynamics: Study on reduction in yarn hairiness and the role of air drag forces and angle of impact of air current. Text. Res. J..

[22] Rengasamy R.S., Kothari V.K., Patnaik A., Punekar H. (2006). Airflow simulation in nozzle for hairiness reduction of ring spun yarns. part I: Influence of airflow direction, nozzle distance, and air pressure. J. Text. Inst..

[23] Wang X., Miao M., How Y. (1997). Studies of JetRing spinning, part I: Reducing yarn hairiness with the JetRing. Text. Res. J..

[24] Cheng K.P.S., Li C.H.L. (2002). JetRing spinning and its influence on yarn hairiness. Text. Res. J..

[25] Qiu H., Zhang Y., Xu Z.L., Ge M.Q. (2012). A novel method to reduce hairiness level of ring spun yarn. Fibers Polym..

[26] Basal G., Oxenham W. (2006). Effects of some process parameters on the structure and properties of vortex spun yarn. Text. Res. J..

[27] Kuppers S., Muller H., Ziegler K., Heitmann U., Planck H. (2008). Spinning limits at vortex spinning at the processing of 100% cotton. Melliand Int..

[28] Ortlek H.G., Ulku S. (2005). Effect of some variables on properties of 100% cotton vortex spun yarn. Text. Res. J..

